# A Polymorphic Gene within the Mycobacterium smegmatis *esx1* Locus Determines Mycobacterial Self-Identity and Conjugal Compatibility

**DOI:** 10.1128/mbio.00213-22

**Published:** 2022-03-17

**Authors:** R. R. Clark, P. Lapierre, E. Lasek-Nesselquist, T. A. Gray, K. M. Derbyshire

**Affiliations:** a Division of Genetics, Wadsworth Center, New York State Department of Health, Albany, New York, USA; b Bioinformatics Core, Wadsworth Center, New York State Department of Health, Albany, New York, USA; c Department of Biomedical Sciences, University at Albany, Albany, New York, USA; University of Massachusetts—Amherst

**Keywords:** conjugation, mycobacteria, kin recognition, conjugation, *esx1*, horizontal gene transfer

## Abstract

Mycobacteria mediate horizontal gene transfer (HGT) by a process called distributive conjugal transfer (DCT) that is mechanistically distinct from *oriT*-mediated plasmid transfer. The transfer of multiple, independent donor chromosome segments generates transconjugants with genomes that are mosaic blends of their parents. Previously, we had characterized contact-dependent conjugation between two independent isolates of Mycobacterium smegmatis. Here, we expand our analyses to include five independent isolates of M. smegmatis and establish that DCT is both active and prevalent among natural isolates of M. smegmatis. Two of these five strains were recipients but exhibited distinct conjugal compatibilities with donor strains, suggesting an ability to distinguish between potential donor partners. We determined that a single gene, *Msmeg0070*, was responsible for conferring mating compatibility using a combination of comparative DNA sequence analysis, bacterial genome-wide association studies (GWAS), and targeted mutagenesis. *Msmeg0070* maps within the *esx1* secretion locus, and we establish that it confers mycobacterial self-identity with parallels to kin recognition. Similar to other kin model systems, orthologs of Msmeg0070 are highly polymorphic. The identification of a kin recognition system in M. smegmatis reinforces the concept that communication between cells is an important checkpoint prior to DCT commitment and implies that there are likely to be other, unanticipated forms of social behaviors in mycobacteria.

## INTRODUCTION

Horizontal gene transfer (HGT) provides gene flow in the otherwise unchanging genomes of asexually reproducing organisms. HGT is mediated by at least three distinct mechanisms: transduction, transformation, and conjugation (1, 2). Unlike transduction and transformation, conjugation requires direct cell-to-cell contact between two viable, coexisting cells: a donor and a recipient. In classic conjugation models, the donor and recipient cells are genetically defined by the presence and absence, respectively, of a conjugative plasmid. DNA transfer is unidirectional, from the plasmid-containing donor to the plasmid-free recipient. The donor cell drives this process, as the plasmid encodes all of the protein products required for transfer, including those for mating-pair formation and DNA processing. The resulting transconjugant acquires the plasmid and now becomes a donor. Thus, DNA transfer is a replicative process and accounts for the rapid spread of the conjugative plasmid through a population of recipient cells. Transfer does not occur between two donors because they exhibit immunity, which is mediated by surface and entry exclusion proteins, such as TraS and TraT in the F plasmid (3). Entry exclusion is a common feature of conjugative plasmids, which is thought to increase donor fitness and prevent lethal zygosis resulting from multiple donor transfer events in a single recipient (4).

We have demonstrated that strains of Mycobacterium smegmatis conjugate by a mechanism that is distinct from the canonical *oriT*-mediated plasmid transfer (5–7). Instead, chromosomal DNA is transferred, which occurs without plasmids or conserved genes known to mediate transfer. The most striking feature of mycobacterial conjugation is that transconjugant chromosomes are mosaics of their parental chromosomes (8). Consequently, we have termed this phenomenon distributive conjugal transfer (DCT) to distinguish it from *oriT*-mediated transfer and to emphasize that the recipient acquires multiple, noncontiguous segments of chromosomal DNA from the donor. These segments are exchanged with homologous recipient sequences around the chromosome with no obvious regional biases. Thus, even though transconjugants can acquire large amounts of DNA from the donor, there is not necessarily a net gain of chromosomal DNA. Therefore, DCT is nonreplicative, and only a subset of the resulting transconjugants become donors.

**FIG 1:**
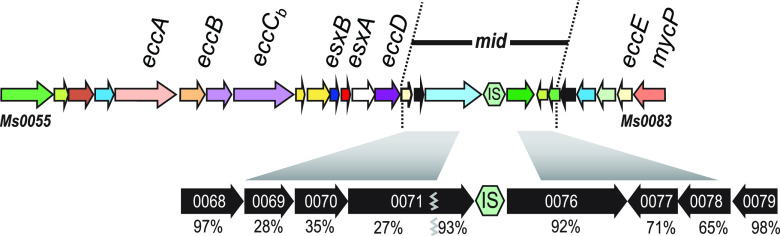
Genetic map of the *mid* locus within *esx1* of the donor strain mc^2^155. The *mid* region of the donor strain M. smegmatis mc^2^155 was defined by linkage mapping to *Msmeg0069–0078* (8). Conserved core *esx* genes, homologs of which are typically found in *esx* loci, are shown above the locus. The encoded core and other Esx proteins are highly conserved (>96.6% amino acid identity), in contrast to the Mid proteins; percentages below the encoding *mid* genes indicate amino acid sequence identities between donor (mc^2^155) and recipient (MKD8) orthologs. Msmeg0071 is poorly conserved at its N terminus (27%), but its C terminus is highly conserved (93% amino acid identity).

Like plasmid transfer, DCT requires direct cell-cell contact and is unidirectional from a donor to a recipient cell (9). However, the genetic basis for donor or recipient ability has not been defined. In our work to date, donor and recipient abilities have been determined empirically. We previously established that 1 to 10% of marker-selected transconjugants become donors (7). Genome sequence analysis determined that there were thousands of SNVs (single nucleotide variants) between our prototype donor and recipient strains (∼1 per 100 nucleotides [nt]). We took advantage of these two observations to generate a linkage map of a donor-conferring locus in the F1 transconjugants using the parent-identifying SNVs (8). We further refined our mapping by backcrossing independent F1 lineages with the original recipient strain while screening transconjugant progeny for donor ability at each successive iteration. This bacterial genome-wide association study (GWAS) approach delimited a region within the *esx1* secretion system locus that conferred mating proficiency and was termed *mid* (for mating identity). *mid* was mapped to a region roughly spanning mc^2^155 donor genes *Msmeg0069* to *Msmeg0078* (*Msmeg0069*–*0078*), based on sequences that were present or absent in different donor- and recipient-proficient recombinants (Fig. 1). The *mid* genes are located within the M. smegmatis *esx1* locus, and mutant derivatives of these genes phenocopy other *esx1* mutants; donor *esx1* mutants are hyperconjugative, while recipient *esx1* mutants are defective in transfer (10, 11).

The precise gene(s) responsible for conferring donor proficiency in our *mid* genetic mapping approach has not been determined. Moreover, our *mid* analyses were based solely on two strains of M. smegmatis, and it is not known whether comparable *mid* loci are conserved and functionally similar in other DCT-proficient M. smegmatis isolates. Here, we show, using mutant derivatives, genetic crosses, and comparative sequencing, that *Msmeg0070* confers strain self-identity, and its expression results in DCT incompatibility with “kin” strains. The presence of polymorphic *Msmeg0070* in many environmental mycobacteria suggests that kin identification is important in promoting beneficial gene flow in mixed mycobacterial communities.

## RESULTS

### Driving recombination within the *esx1* locus by DCT further refines *mid*.

Our standard pair of conjugal M. smegmatis strains are antibiotic-resistant derivatives of mc^2^155 (the common laboratory strain) and MKD8 (a streptomycin [Str]-resistant independent isolate), which have been the focus of most of our studies (9). We previously defined mc^2^155 as a conjugal donor and MKD8 as a recipient in this cocultured pairing. The acquisition of the *mid* locus genes from a donor strain (mc^2^155) converted the recipient strain (MKD8) to a conjugal donor; this transconjugant could now cross with a second MKD8 recipient. That approach initially mapped the locus to *Msmeg0069*–*0078*, comprising six unique protein-coding genes and a cluster of insertion sequences (ISs) mapping within the *esx1* locus (8). We have since further refined *mid* to three genes, *Msmeg0069*–*0071*, by a similar GWAS approach, using parental derivatives that had selectable markers either directly flanking or within *esx1* (data not shown). These three *mid*-encoded proteins are more dissimilar between mc^2^155 and MKD8 (28, 35, and 27% amino acid identities, respectively) (Fig. 1) than the three now-excluded *mid* genes (92, 71, and 65% amino acid identities for Msmeg0076–0078, respectively) and all other *esx1*-encoded proteins (>96.6% amino acid identity). The lack of DNA sequence conservation in this region prevented further dissection by DCT (RecA)-mediated recombination.

In an alternative approach described here, we have determined the sequence of the *esx1* regions for three additional environmental isolates of M. smegmatis and compared the proteins encoded in their *mid* loci to correlate *mid* gene content with their pairwise mating phenotypes.

### Environmental M. smegmatis genomes are mosaic, consistent with their generation by DCT.

**FIG 2:**
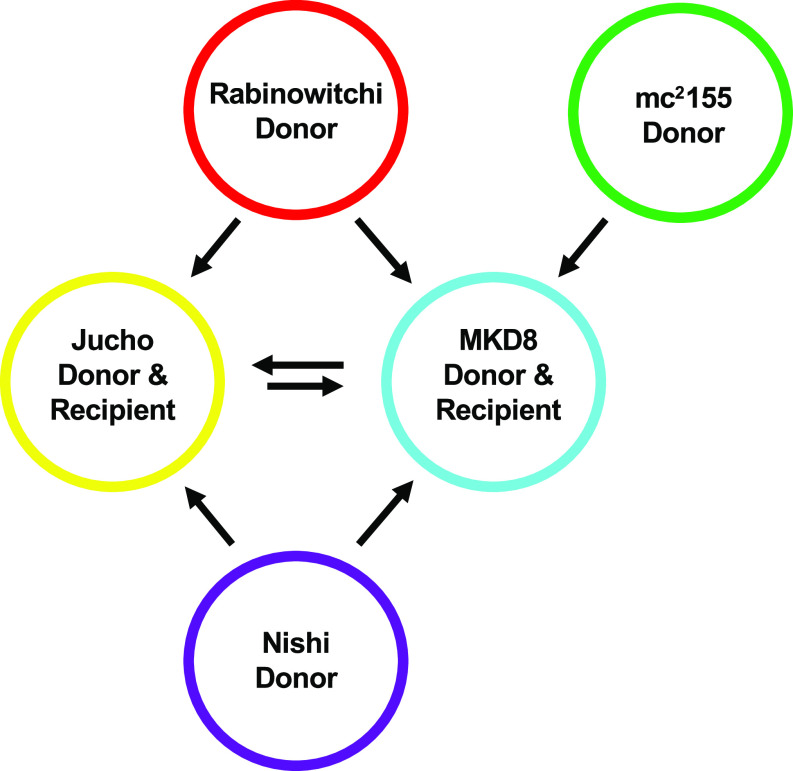
Summary of interstrain mating that occurs between independent environmental isolates of M. smegmatis (9 and 13). Arrows indicate the directionality of transfer, from donor to recipient. Jucho and MKD8 act as recipients in cocultures with donor-only strains, but they can exchange DNA bidirectionally when cocultured together, indicating that they can also act as donors.

In addition to our standard pair of conjugal M. smegmatis strains, three other isolates of M. smegmatis had been shown to generate recombinants in specific, pairwise crosses (Fig. 2) (9, 12, 13). Two of these isolates (Rabinowitchi and Nishi) behaved as donors when paired with either of the recipient strains, MKD8 or the third isolate, Jucho. In total, three strains (mc^2^155, Rabinowitchi, and Nishi) behaved exclusively as donors; marker transfer was never detected between them and was always unidirectional (donor to recipient) (9). Remarkably, the two recipient strains, Jucho and MKD8, also exchanged DNA bidirectionally when cocultured; i.e., they can behave as either a donor or a recipient with each other (Fig. 2). The observation that each of these natural isolates generated dual-antibiotic-resistant recombinants in specific pairwise cocultures was consistent with DCT.

**FIG 3:**
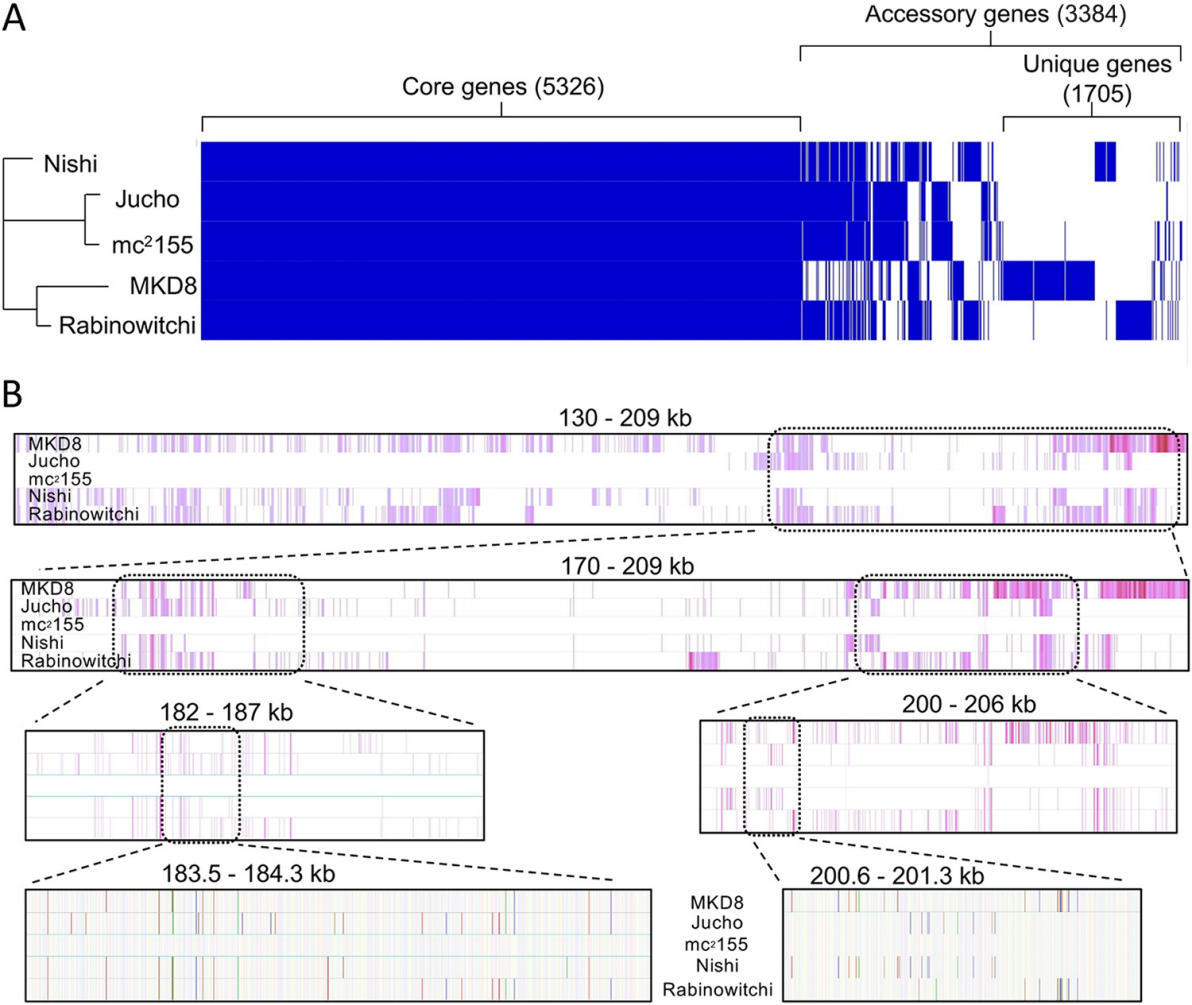
Genome comparisons of the five independent environmental isolates of M. smegmatis. (A) Pangenome analysis identifies conserved core genes among the M. smegmatis isolates. Roary v.3.13.0 (42) was used to perform a pangenome analysis with default settings. Orthologs were required to share at least 95% global amino acid identity and are indicated by a vertical blue line in each genome along the *x* axis (the pangenome); the white (no blue) line indicates that an ortholog of >95% identity is absent in that genome. Clustered are conserved core genes (left), accessory genes (middle right), or lineage-specific genes (right) found in only one of the five strains. Results were visualized with Phandango v.1.3.0 (43) and Figtree v.1.4.4 (https://github.com/rambaut/Figtree). (B) A multiple alignment of the five mycobacterial genomes was performed with Parsnp and visualized using Gingr (37) to show collinearity and SNVs in a 79-kb region spanning nt coordinates 130000 to 209000 in the mc^2^155 genome. In the upper panels, vertical purple bars indicate SNVs present in each genome, using mc^2^155 as the reference sequence, which is depicted in the middle row as a white bar. Consecutive panels zoom in on the indicated regions. The bottom panel is at single-nucleotide resolution, and the colors correspond to GCAT (yellow, blue, green, and red, respectively). Bold colors are SNVs, and faintly colored vertical bars match the mc^2^155 sequence.

To fully utilize these isolates as parental strains in DCT experiments and to determine if DNA transfer resulted in mosaic genomes, we first determined their genome sequences. The sequences were assembled *de novo*, resulting in closed, single-contig genomes (Table 1). None of the five M. smegmatis strains harbor naturally occurring plasmids, which are commonly found in conjugative bacteria. Each of the finished genomes was similar in length and overall collinearity. Pangenome analysis revealed that there were 5,326 core genes (>95% identity in all five strains) and 3,384 accessory genes (>95% identity in fewer than five strains) (Fig. 3A).

**TABLE 1:** M. smegmatis genome sequence summary^*a*^

M. smegmatis strain	Genome length (Mb)	No. of predicted genes	No. of predicted unique genes	GenBank accession no.
MKD8	7.119169	6,755	818	CP027541.1
Jucho	6.895172	6,558	34	CP080274
Rabinowitchi	7.061747	6,709	386	CP080272
Nishi	7.010278	6,673	275	CP080273
mc^2^155	6.988209	6,681	140	NC_008596

a Gene predictions and annotations were performed using Prokka (36). The number of unique genes was provided by a pangenome analysis using Roary (42). Predicted unique genes are those genes found in only one of the five strains. Genes in common between strains and unique genes are listed in Table S1 in the supplemental material.

10.1128/mbio.00213-22.3 TABLE S1 List of shared and unique genes among five M. smegmatis isolates predicted by pangenome analysis in Roary (42). Each gene name or orthologous group is listed with the size range (base pairs) and the strain(s) in which the gene is found. Genome annotations were performed using Prokka (36). Download Table S1, XLSX file, 0.7 MB. Copyright © 2022 Clark et al. 2022 Clark et al. https://creativecommons.org/licenses/by/4.0/ This content is distributed under the terms of the Creative Commons Attribution 4.0 International license.

While a gene-level analysis provides a broad overview of genome diversity, DNA transferred by DCT can be tracked more specifically by lineage-specific SNVs. Nucleotide-level analyses identified large numbers of indels and SNVs when the genomes of M. smegmatis isolates were compared with each other and with the mc^2^155 reference genome (Table 2). Pairwise comparisons between most of the isolates identified >40,000 SNVs, while Jucho and mc^2^155 were the most closely related, with just 7,456 SNVs (averaging ∼1 per kb).

**TABLE 2:** SNVs identified by pairwise comparisons between M. smegmatis strains^*a*^

Strain	No. of SNVs				
	Jucho	mc^2^155	MKD8	Nishi	Rabinowitchi
Jucho	0	7,456	66,355	40,165	39,869
mc^2^155		0	65,381	41,076	41,077
MKD8			0	68,595	68,328
Nishi				0	35,620
Rabinowitchi					0

a SNVs were identified by snp-dists (https://github.com/tseemann/snp-dists) and are limited to orthologous coding sequences.

Genomic alignments produced by SibeliaZ allowed us to assess the degrees of diversity and recombination among our five isolates (14). This analysis showed that the five M. smegmatis genomes, while collinear, were highly mosaic at both the gene and nucleotide levels (see Table S1 in the supplemental material). Mosaicism extends across all the isolates and is easily visualized in multiple-sequence alignments by multiple blocks of nucleotide diversity (depicted as purple vertical bars in Fig. 3B, top) sandwiched between identical sequences (white bars). Figure 3B shows a comparison of a 79-kb region for all five strains, in which consecutive panels progressively zoom in to the nucleotide level. There are large regions of shared nucleotide identity and gene collinearity, which are uninformative for documenting segment exchange. However, these shared regions are interrupted by segments of DNA that, while very similar, contain distinguishing SNVs. Some of these SNV-intensive regions are shared between two or more isolates, suggesting either vertical transmission from a recent common ancestor or a horizontal exchange of DNA. Other regions of diversity are unique to a strain, suggesting that they are lineage specific or were acquired from other environmental mycobacteria that have not been sequenced. The extensive mosaicism evident in these genomes shows that horizontal gene transfer is a major force in shaping them and is consistent with DCT occurring in natural M. smegmatis communities.

### Conjugal DNA transfer between independent isolates of M. smegmatis generates progeny with mosaic genomes.

We applied our DCT experimental protocol to pairwise cocultures of chromosomally marked donor and recipient derivatives of the five isolates to generate independent, dual-antibiotic-resistant transconjugants. Single transconjugant colonies were picked and restruck onto dual-antibiotic plates to ensure clonal purity before the isolation of genomic DNA for whole-genome sequencing. Twenty-three transconjugant genomes were sequenced and assembled by alignment to their parental reference genomes.

**FIG 4:**
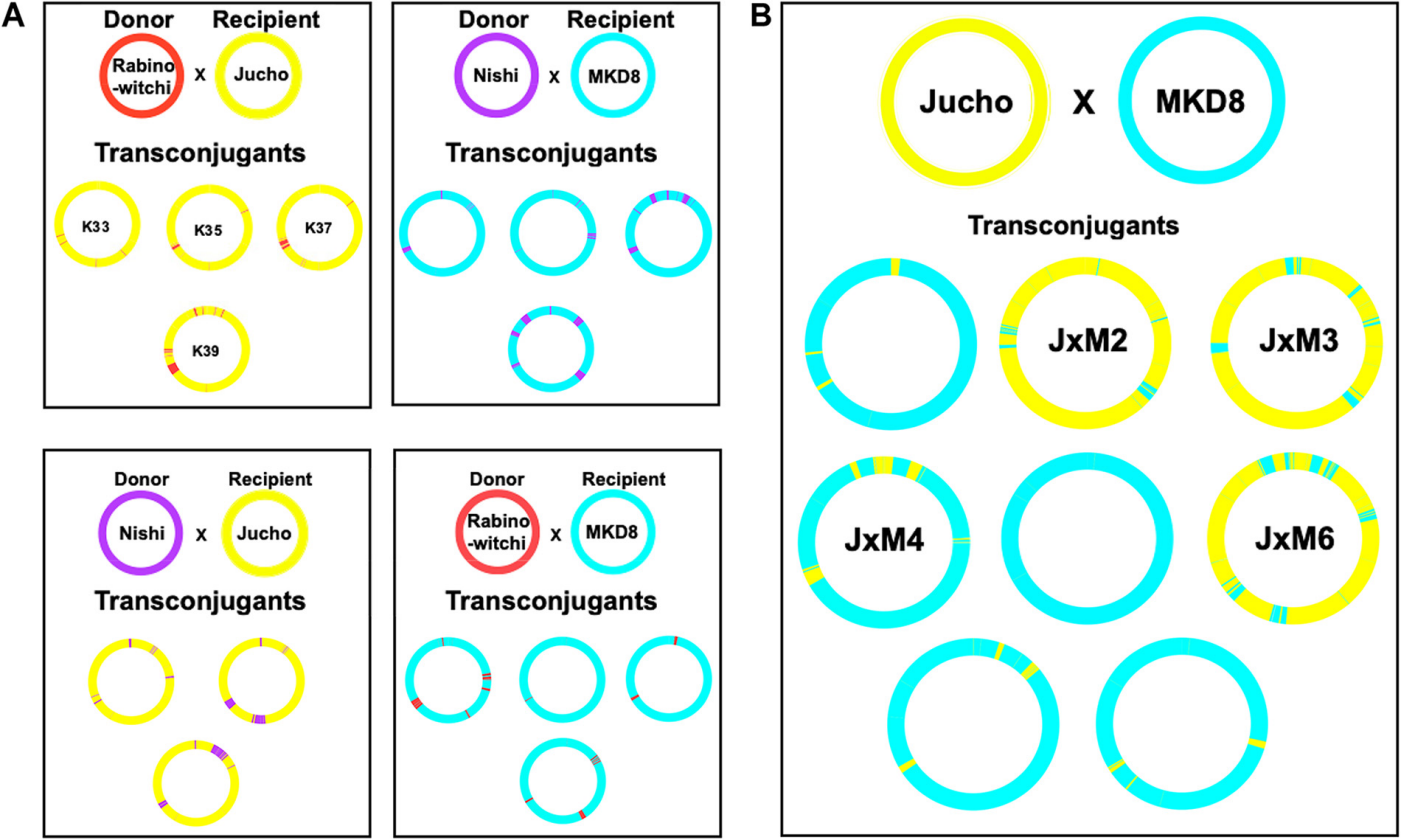
Circos plots showing the distribution of parental DNA in sequenced transconjugants. (A) Crosses between defined donor and recipient strains in which transfer is unidirectional. (B) Transconjugant progeny of MKD8 and Jucho strains in which DNA transfer is bidirectional. Each parent genome is color-coded (as in Fig. 2) to indicate the source and visualize the distribution of transferred DNA in the transconjugant genomes. All crosses are between a Km-resistant donor and an Str-resistant recipient. The Km gene is inserted at the *attL5* locus at ∼8 o’clock on the chromosome. Transfer of this DNA segment can be seen against the recipient background in most crosses. In a few examples, the transferred segment was too small to be resolved in the Circos plot.

The known SNVs inherited from each parental genome distinguish transferred segments, and the relative proportion from each parent indicates the directionality of DNA transfer. The resulting transconjugant genomes were mosaic as revealed by the genome-wide distribution of segments of DNA having donor-specific SNVs (Fig. 4A) embedded in a recipient-specific SNV background. Regions of recombination varied among transconjugant genomes produced by a single pair of parents, indicating that the progeny are independent while also underscoring the instantaneous, genome-wide diversifying potential of DCT. Transfer between all conjugation-proficient pairs was unidirectional, producing transconjugant genomes that were primarily composed of DNA from the recipient (either MKD8 or Jucho), punctuated by tracts of DNA from the paired donor (mc^2^155, Rabinowitchi, or Nishi) (Fig. 4A and Table 3). The percentage of transferred donor DNA ranged between 1 and 12%, similar to the range observed in crosses between mc^2^155 and MKD8 (8), and these were mediated by an average of 16 recombination events. Together, these data establish that DCT is active among natural isolates of M. smegmatis.

**TABLE 3:** Analysis of transconjugant genomes^*a*^

% parental DNA	% parental DNA	No. of transferred regions^*b*^	Largest region (bp)
MKD8	Jucho		
98.41	1.30	6	54,500
4.33	91.46	23	80,500
6.21	89.88	22	132,500
87.09	10.79	15	185,000
99.52	0.04	4	1,000
13.63	81.31	32	163,500
95.19	4.26	10	93,500
96.75	2.83	10	80,000
Recipient	Donor		
MKD8	Nishi		
97.30	3.22	27	92,500
98.01	2.58	10	43,000
90.97	8.36	13	166,500
87.73	12.49	14	221,000
Recipient	Donor		
Jucho	Nishi		
97.17	3.47	36	65,000
90.93	9.62	28	233,500
90.69	9.06	19	146,000
Recipient	Donor		
MKD8	Rabinowitchi		
91.49	8.03	16	128,000
99.27	0.31	3	18,500
96.66	2.31	4	85,000
94.97	4.24	11	118,000

Recipient	Donor		
Jucho	Rabinowitchi		
97.84	1.16	11	21,000
97.07	2.05	12	76,500
95.15	3.95	16	120,500
91.32	7.85	18	260,500

a The percentage of parental DNA found in each transconjugant is listed, along with the number of segments transferred into the recipient and the size of the largest segment of transferred DNA in base pairs.

b The algorithm used for determining the number of regions transferred used nonoverlapping windows of 500 bp (39). Thus, small rearrangements of <500-bp segments are not counted in the number of events.

We note that the colony morphology of the transconjugants varied, even between transconjugants generated from the same parental cross, suggesting that these colony variants were a result of the shuffling of alleles embedded in cell wall biosynthetic genes and/or their regulators (Fig. S1). Thus, even in this small sample set, it was evident that DCT has the ability to create new genetic combinations with clear phenotypic consequences.

10.1128/mbio.00213-22.1 FIG S1 Morphotypes of transconjugants from a cross between MKD8 and Jucho. Despite being generated from identical parents, these F1 transconjugants have very distinct colony morphologies, most likely resulting from novel, cell-wall-gene mosaics generated by DCT. The morphotype of JxM1 is similar to that of MKD8, while the morphotype of JxM4 resembles that of Jucho despite having a predominantly MKD8-like genome. Download FIG S1, PDF file, 3.0 MB. Copyright © 2022 Clark et al. 2022 Clark et al. https://creativecommons.org/licenses/by/4.0/ This content is distributed under the terms of the Creative Commons Attribution 4.0 International license.

### The two recipient strains are capable of bidirectional DCT.

Jucho and MKD8 behave as recipients when cocultured with dedicated conjugal donor strains. However, previous studies observed bidirectional chromosome marker transfer between these two isolates although at ∼10-fold-lower frequencies than in unidirectional donor-recipient crosses (9). Additional nonselectable markers showed that each strain could act as a donor. Thus, Jucho and MKD8 are both dedicated recipients and conditional donors. Genome analysis of eight transconjugants obtained from crosses between Jucho and MKD8 identified two distinct classes, consistent with each strain acting as either a donor or a recipient (Fig. 4B). The genome of each transconjugant was predominantly (81 to 99%) retained from one of the two parent genomes, with the balance of DNA being acquired horizontally (Table 3). Three of the transconjugant genomes were composed primarily of Jucho (yellow) DNA, and five had an MKD8 (cyan) genetic background (Fig. 4B).

### Recipient transconjugants can switch donor-selective phenotypes.

Jucho and MKD8 have differing specificities with respect to donor strain compatibility; while both strains act as recipients when cocultured with a donor, Rabinowitchi (or Nishi), Jucho does not produce transconjugants when mated with mc^2^155 (Fig. 2). Given the mosaicism observed in the Jucho-MKD8 transconjugant genome sequences, we hypothesized that some transconjugants may have switched their donor compatibility. We tested the four transconjugants with the most mosaic, and, therefore, most likely informative, genomes for altered conjugal selectivity. JxM2 (to indicate the second transconjugant evaluated from the coculture of Jucho and MKD8), JxM3, and JxM6 have predominantly Jucho genomes, while JxM4 has a more MKD8-like genome. Each transconjugant was tested for its ability to cross with a differently marked, antibiotic-resistant strain. Two of the transconjugants had switched donor compatibility relative to the predominant parental genomic content (Table 4). JxM3 had acquired the ability to mate proficiently with mc^2^155 despite having a predominantly Jucho genome and retaining conjugal compatibility with Rabinowitchi. When mated with either of its parental strains, JxM3 was conjugal with Jucho but not with MKD8. Therefore, the conjugal compatibility profile of JxM3 matched that of MKD8. Conversely, JxM4 had the conjugal compatibility profile of Jucho despite having a predominantly MKD8 genome: it produced transconjugants with Rabinowitchi and MKD8 but not with mc^2^155 or Jucho. The JxM2 and JxM6 strains retained the conjugal profile of their Jucho recipient parent strain; they were transfer proficient with Rabinowitchi but failed to produce transconjugants with mc^2^155 or Jucho (self).

**TABLE 4:** Transconjugants with altered donor specificity^*a*^

Donor strain	Result of cross with recipient strain			
	MKD8	Jucho	JxM3	JxM4
mc^2^155	+	−	+	−
Rabinowitchi	+	+	+	+
JxM3	−	+	ND	+
JxM4	+	−	ND	ND

a Results of pairwise crosses between differently marked donor and recipient strains are shown. “+” indicates transconjugants obtained at normal frequencies (∼1 × 10^−4^ per donor). “−” indicates that no or very few transconjugants were obtained (<1 event/10^9^ donors). JxM3 and JxM4 are two independently isolated transconjugants from a cross between Jucho and MKD8, and their mosaic genomes are depicted in Fig. 4B. ND, not done. The JxM4 × JxM3 cross was not done as it is the reciprocal cross to JxM3 × JxM4.

The most parsimonious explanation for the switched donor specificity of JxM3 and JxM4 is that they have exchanged reciprocal sets of homologous parental genes that determine donor selectivity for recipients. JxM3 is predominantly of Jucho origin (93%) but had two large regions exchanged for MKD8 DNA. Most notable is a 164-kb region spanning the origin of replication and the *esx1* locus (up to MKD8 coordinate 129151) (Fig. 4B). In contrast, JxM4 had acquired the *esx1* region from Jucho, while most of the rest of its genome is of MKD8 origin (89%). Therefore, these data independently and reciprocally implicate the *esx1* region as the determinant of donor compatibility with a recipient, extending our previous findings from mc^2^155 and MKD8 to other natural isolates of M. smegmatis (8). In the experiments described below, we show that homologs of *Msmeg0070*, within the *mid* locus of *esx1*, are responsible for determining self-identity and, thereby, DCT compatibility.

### Mating (in)compatibility is mediated by *mid*.

**FIG 5:**
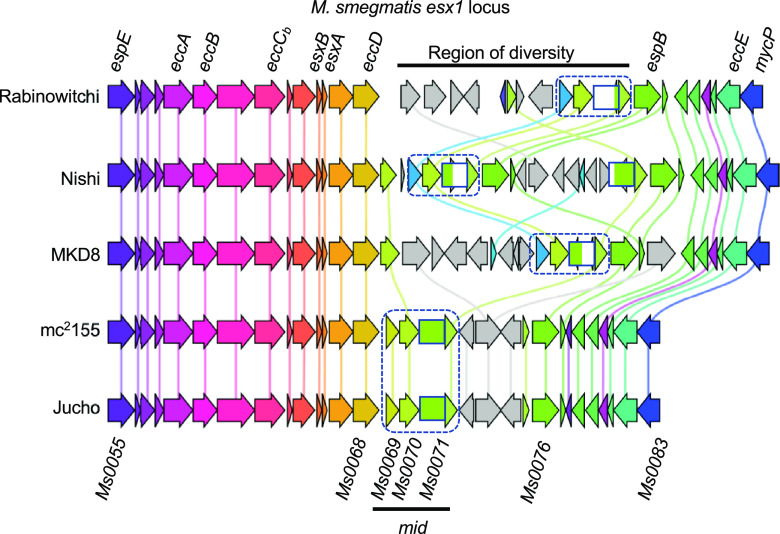
Comparative gene analysis of the *esx1* regions of the five M. smegmatis genomes. The schematic shows the overall collinearity and conservation of *esx1* while highlighting the diversity of the *mid* region. The non-*mid esx1* genes (*Msmeg0055–0068* and *Msmeg0076–0083*) encode highly conserved proteins (>96.6% amino acid identity). However, proteins encoded by genes between these regions are poorly conserved (<30% amino acid identity) and include gene rearrangements, duplications, and multiple insertion sequence (IS) elements (*mid* genes within this region are boxed [*Msmeg0069–0071*]). Remarkably, mc^2^155 and Jucho are identical throughout the region, with 2 nucleotide differences from mc^2^155 in *Msmeg0067* (resulting in an Arg-to-Pro amino acid change) and *eccE* (a silent C-to-T nucleotide substitution). Comparisons of *esx1*-encoded proteins were generated by Clinker using a best-BLAST-hit approach to identify orthologous genes at the 5′ and 3′ ends of each region (44). Orthologous genes are color-coded, and ISs and remnants of ISs are shaded in gray. Vertical lines drawn in the same color connect homologs. Note that the low amino acid conservation in the N terminus of Msmeg0071 prevented Clinker from identifying the complete gene, which we indicate here with a green striped box. Similarly, the low conservation of *Msmeg0069* resulted in two classifications, identical in mc^2^155 and Jucho (olive arrow) and related but depicted in blue in Rabinowitchi, Nishi, and MKD8, immediately upstream of *Msmeg0070–0071* orthologs. The names of conserved *esx* genes are indicated at the top of the alignment, and M. smegmatis numerical gene identifiers are shown at the bottom for reference.

Sequence comparisons of the parental isolates had indicated that they were highly mosaic (Fig. 3). Across the group, the core ESX-1 (non-Mid) proteins are well conserved at the amino acid level (>96.6% identity), with hundreds of SNVs being scattered throughout the locus (Fig. 5 and Table S2). In contrast, the *mid* regions differ markedly between strains, with gene rearrangements, duplications, and additional gene paralogs. Remarkably, we noted that the *esx1* regions of mc^2^155 and Jucho were essentially identical at the nucleotide level (only 2 SNVs in the entire >33-kbp *esx1* locus and no changes within *mid*), suggesting recent acquisition or shared ancestry (Fig. 5). The remarkable *mid* gene identity between Jucho and mc^2^155 suggested a basis for their exceptional inability to conjugate: the identical *mid* regions conferred identity of self, which may preclude conjugal compatibility. This is also consistent with the absence of conjugation observed in differentially marked same-strain cocultures that otherwise demonstrate bidirectional transfer (Jucho × Jucho or MKD8 × MKD8). Thus, donor and recipient pairs with identical *mid* regions are incompatible, but those pairs with different *mid* regions are transfer proficient.

10.1128/mbio.00213-22.4 TABLE S2 Pairwise comparison of *esx1* single nucleotide polymorphisms (SNPs). The two SNVs identified between Jucho and mc^2^155 are a G-to-C transversion at position 87853 located in *Msmeg0067*, which creates an Arg-to-Pro substitution and a silent C-to-T transition in *eccE*. Download Table S2, DOCX file, 0.02 MB. Copyright © 2022 Clark et al. 2022 Clark et al. https://creativecommons.org/licenses/by/4.0/ This content is distributed under the terms of the Creative Commons Attribution 4.0 International license.

**FIG 6:**
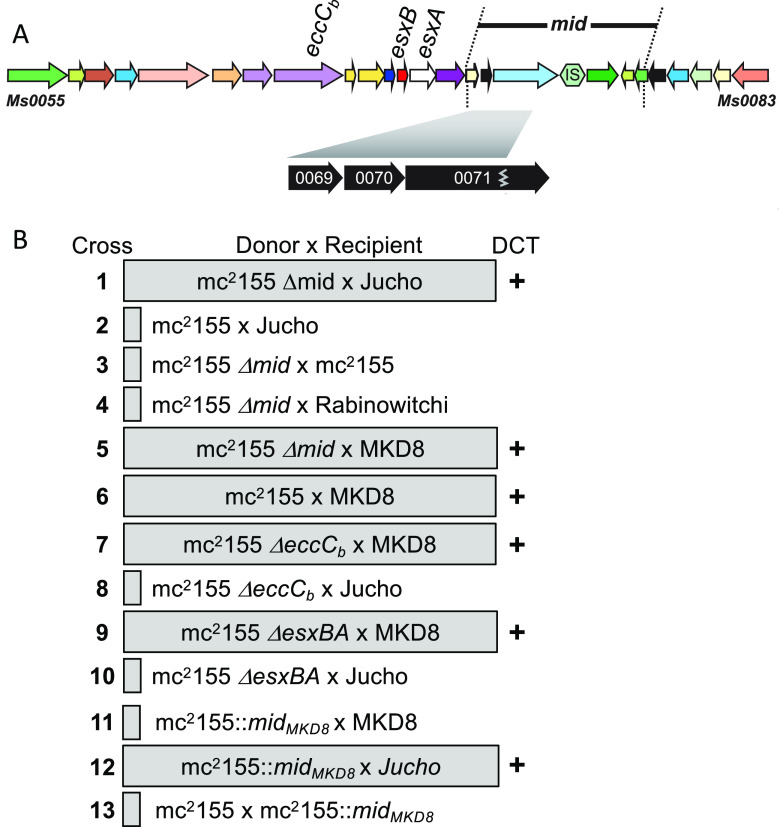
*mid* confers self-identity and determines mating compatibility. (A) Map of *esx1* and *mid* indicating the relevant genes used in the study. (B) Mating pairs boxed inside the shaded rectangle generated transconjugants at high frequencies (>1 × 10^−4^ per donor) (DCT “+”). Unboxed mating pairs produced no or very few transconjugants (<1 × 10^−9^ donors). In all crosses, parent donor and recipient counts were similar.

We hypothesized that *mid* gene expression produces proteins that identify a bacillus as kin. To begin to test this, we created a precise deletion of the originally defined mc^2^155 donor *mid* region (*Msmeg0069*–*0078*) (mc^2^155 Δ*mid*). mc^2^155 Δ*mid* transferred at high frequencies (1 × 10^−4^) with Jucho, in contrast to the wild-type mc^2^155 donor (<1 × 10^−9^) (Fig. 6B, rows 1 and 2). Thus, the deletion of *mid* from mc^2^155 made it transfer proficient with Jucho. This gain of donor proficiency to include Jucho was not a reversal of the conjugal role: mc^2^155 Δ*mid* retained both the inability to conjugate with the donors Rabinowitchi and mc^2^155 (self) and conjugal compatibility with MKD8 (Fig. 6B, compare rows 3 to 5 with the wild-type control in row 6). We further established that this phenotype was a consequence of the *mid* deletion and not a disruption of ESX-1 function by showing that deletions of mc^2^155 *esx1* genes, *eccC*_b1_ or *esxBA*, did not confer compatibility with Jucho, yet these mutants were still transfer proficient with MKD8 (Fig. 6B, rows 6 to 10). Collectively, these data show that it is the loss of the *mid*-encoded function and not the ESX-1 secretion function that enabled the conjugal compatibility of mc^2^155 Δ*mid* with Jucho.

To further test the role of *mid*, we modified the mc^2^155 donor genome to express the *mid* region from MKD8 (*mid*_MKD8_) (genes *MKD8_0069*–*0071*). Allele exchange was used to precisely replace the mc^2^155 *mid* genes *Msmeg0069*–*0071* with *mid*_MKD8_ to create mc^2^155::*mid*_MKD8_. No transconjugants were generated in crosses between mc^2^155::*mid*_MKD8_ and MKD8 in a standard DCT assay (Fig. 6B, row 11). This outcome is consistent with the *mid*_MKD8_ genes, now expressed in both donor and recipient, determining “self” and preventing conjugal compatibility. Further supporting the model, this *mid*-switched mc^2^155 strain was transfer proficient with Jucho, indicating that Jucho and mc^2^155::*mid*_MKD8_ no longer exclude each other and that mc^2^155::*mid*_MKD8_ remained donor proficient despite encoding a recipient-derived *mid* locus (Fig. 6B, row 12). Finally, we showed that mc^2^155::*mid*_MKD8_ has not been converted to a recipient, as no DCT was detected in coculture with a differentially marked mc^2^155 (Fig. 6B, row 13). The latter result underscores that being a recipient is a property of MKD8 and Jucho and that recipient ability is not mediated by *mid*.

### Msmeg0070 determines self-identity.

**FIG 7:**
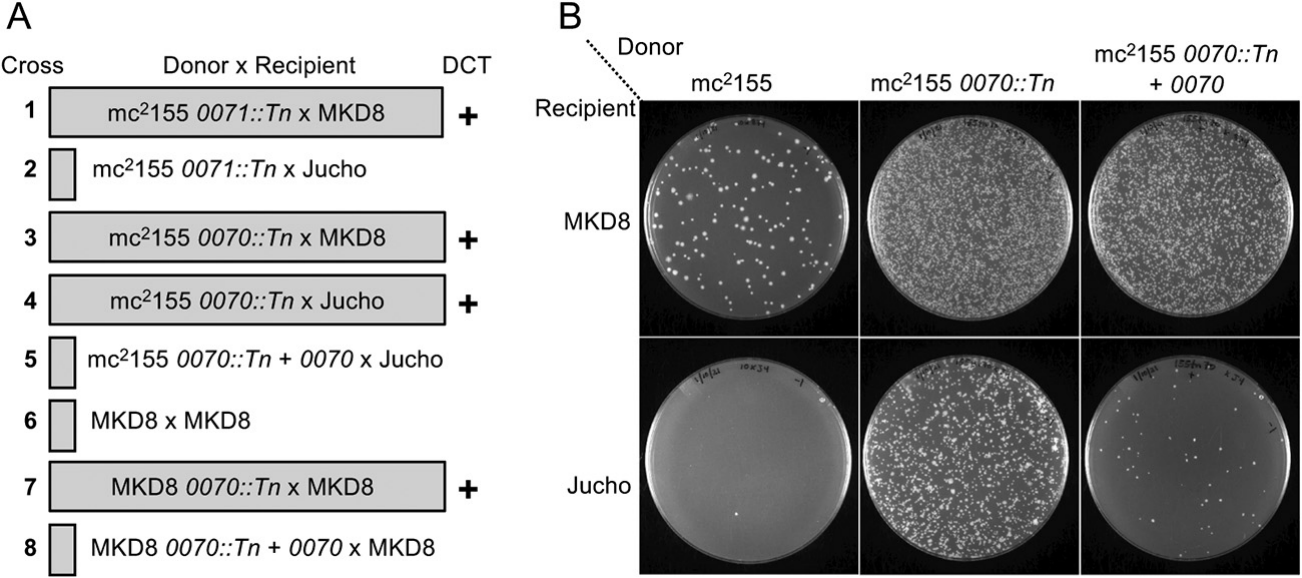
*Msmeg0070* confers self-identity. (A) Mating pairs used in the analysis and their transfer proficiency. Pairs boxed inside a shaded rectangle were transfer proficient (DCT +). Unboxed mating pairs produced no or very few transconjugants (<1 × 10^−9^ donors). In all crosses, viable donor and recipient counts were similar. (B) Image of transconjugant-selective plates at a 10^−1^ dilution for the donor-recipient pairs indicated. Crosses between mc^2^155 and Jucho are nonproductive (bottom left plate), but disruption of *Msmeg0070* in mc^2^155 results in high transfer frequencies, which can be suppressed by the ectopic expression of *Msmeg0070*. Note the hyperconjugative phenotype of the *Msmeg0070* mutant with MKD8 (top, compare the left and middle plates), which is also partly suppressed by expressing *Msmeg0070* (right plate).

Transposon (Tn) insertions in many mc^2^155 *esx1* genes were previously identified by their hyperconjugative phenotype with MKD8, including insertions in *Msmeg0070* and *Msmeg0071* (11). We tested these *mid* gene transposon mutants for conjugal compatibility with Jucho. The insertion in mc^2^155 *Msmeg0071* had no effect on its conjugal compatibility; it produced transconjugant progeny only upon coculture with MKD8. However, transposon disruption of mc^2^155 *Msmeg0070* allowed productive transfer with both Jucho and MKD8 (Fig. 7A, rows 1 to 4). Ectopically expressing a wild-type copy of *Msmeg0070* from a plasmid suppressed the ability of the mc^2^155 *Msmeg0070*::Tn donor to conjugate with Jucho (Fig. 7A, compare rows 4 and 5, and Fig. 7B). This complementation further confirmed that *Msmeg0070* (and not potential polar effects on *Msmeg0071*) was responsible for the exclusion of Jucho as a conjugal partner.

Transposon insertion mutants of MKD8 had previously identified *MKD8_0070* as one of many *esx1* locus genes required in the recipient for conjugation (10). The DCT compatibility of two recipient strains (Jucho and MKD8) that have different *mid* regions suggests that donor ability is a general feature secondary to *mid* compatibility. We therefore hypothesized that while the *MKD8_0070*::Tn strain cannot function as a recipient, its loss of identity and inherent donor ability would allow conjugal pairing with itself, MKD8. We tested this and found that disruption of *MKD8_0070* allowed self-mating of MKD8 (Fig. 7A, compare rows 6 and 7). The introduction of a plasmid expressing *MKD8_0070* restored the suppression of self-mating (Fig. 7A, row 8). Together, these data support a working model in which *Msmeg0070* confers self-identity and this determines the compatibility of donor-recipient pairs for DCT.

## DISCUSSION

### Mosaicism is consistent with DCT in environmental isolates.

The extensive mosaicism evident in the genomes of naturally occurring M. smegmatis isolates suggests that horizontal gene transfer is highly dynamic in environmental M. smegmatis communities. Our demonstration of DCT among these isolates to produce transconjugant progeny with mosaic genomes shows that DCT likely contributes to the mosaicism observed in these M. smegmatis genomes. The genomic heterogeneity and mosaicism evidenced in our five isolates predict that extant M. smegmatis is a diverse species and that DCT is active in mixed natural communities; this diversity is only partially encapsulated by our small sampling of five strains.

Evidence for mosaic genomes in other mycobacteria requires closed whole-genome sequence availability. The bias for sequencing clinical specimens likely underestimates mosaicism in mycobacterial genomes, as clinical isolates are often derived from clonal expansions that have had little exposure to other mycobacteria and, thus, the opportunity for HGT. Examples of genomic mosaicism have been observed in the genomes of isolates from the Mycobacterium kansasii complex, M. canettii, and M. abscessus (15, 16). For *M. canettii*, there is more direct evidence for DCT, as not only do natural isolates have mosaic genomes (17), but strains can also be cocultured to produce recombinants with mosaic genomes (18, 19). As we have observed with M. smegmatis, the key in establishing DCT in *M. canettii* and related Mycobacterium tuberculosis complex strains was the identification of *M. canettii* recipient strains, as most strains could act as donors. The contributions of DCT to promoting genetic exchange among other mycobacteria will require a comprehensive sequence analysis combined with experimental approaches using defined parental strains.

### *mid* confers self-identity.

Our previous work had shown that the *mid*_155_ locus, when transferred to an MKD8 recipient, conferred donor ability; the MKD8 *mid*_155_ transconjugant could now mate with a second MKD8 recipient (8). Our interpretation of those data followed standard linkage mapping concepts, that a gene(s) in the *mid* locus conferred donor ability to a recipient. Our new data, encompassing other isolates of M. smegmatis, modify that interpretation and collectively show that the *mid* locus encodes self-identity. Mutating or deleting mc^2^155::*mid*_155_ allowed this donor to be DCT proficient with Jucho, which has a *mid* locus identical to that of mc^2^155 (Fig. 6). Applying this model of *mid* as a self-identity locus to our previous data indicates that MKD8 *mid*_155_ has not acquired donor ability *per se* but, instead, is no longer recognized by MKD8 as kin. Thus, transfer can now occur from this “rebranded” *mid* strain into an MKD8 recipient. Similarly, the inactivation of *MKD8_0070* by a transposon insertion rebranded the strain as “nonkin” to allow a productive cross with MKD8 (Fig. 7). The reciprocal rebranded mc^2^155 *mid* had comparable effects; mc^2^155::*mid*_MKD8_ switched mc^2^155 to be mating compatible with Jucho but mating incompatible with MKD8. Thus, *mid*, and specifically *Msmeg0070* and its orthologs, confers self-identity, which we observe in DCT assays as mating incompatibility.

We suggest that *mid* serves several purposes. It ensures that DCT occurs only between nonkin. *mid* likely serves as a checkpoint for DCT, ensuring the appropriate donor-recipient cell contact prior to the initiation of the DNA transfer process. Such a *mid* checkpoint would prevent commitment to a DCT process that is likely to be energy-intensive or potentially damaging for the participants. If an evolutionary benefit of DCT is to promote genomic diversification, then the neutral exchange of identical chromosomal segments between kin would not be beneficial.

### All strains are donors.

Clarifying the function of *mid* also clarified donor and recipient abilities. The DCT pathways enabled by the rebranding of *mid* between previously incompatible mating pairs (e.g., Jucho and mc^2^155) give a more complete picture of recipient and donor functions. Thus, while the ability to be a recipient is a property of Jucho and MKD8 (Fig. 2), all five strains can function as donors. We now speculate that all strains of M. smegmatis are capable of acting as DCT donors when paired with a *mid*-compatible recipient. The identification of *mid* as a locus of kin exclusion offers an important first step to predict conjugal pairs in M. smegmatis (and potentially other mycobacteria).

### The *mid-esx1* paradox.

The location of *mid* within *esx1* presents a paradox. In pairwise DCT assays, *mid* gene mutants perform exactly as would be predicted of any *esx1* gene: *mid* donor mutants are hyperconjugative, and *mid* recipient mutants cannot receive DNA. However, the function of *mid* in self-identification appears to be independent of ESX-1 function, as *mid* self-identity remained intact in *eccC*_b_ and *esxBA* deletion mutant donors (Fig. 6). Moreover, transposon insertions in the MKD8 *mid* genes *Msmeg0070* and *Msmeg0071* do not affect ESX-1 function as assessed by the continued secretion of the primary secretion substrate, EsxAB (10). Our data here suggest that ESX-1 provides an important supporting role in DCT. The identical *esx1* loci of recipient (Jucho) and donor (mc^2^155) strains clearly show that ESX-1 does not determine recipient or donor ability *per se*, but ESX-1 function is needed to regulate cell-cell responses, presumably by the secretion of different effector proteins (20).

### *mid* genes encode highly polymorphic proteins.

Analysis of transcriptional and translational profiling data indicates that mc^2^155 *Msmeg0070* is misannotated (21). Expression data suggest that the encoded protein is 277 amino acids longer than its predicted length of 111 amino acids (http://smegmatis.wadsworth.org/). Consistent with this, *Msmeg0070* orthologs in MKD8, Jucho, Rabinowitchi, and Nishi are also predicted to encode long proteins (∼380 amino acids) (see Fig. S2 in the supplemental material). The amino acid sequence of the corrected Msmeg0070 protein is extremely proline rich (41/388 residues), with no predicted structural or sequence similarities to other proteins in the NCBI database. Msmeg0070 is predicted to have a transmembrane domain (from residues 67 to 89). It seems logical that a determinant of identity of a contact-dependent process would be present on the extracellular surface of the cell. Localization studies will be required to determine whether Msmeg0070 associates with the mycolate outer membrane, the inner cell membrane, or another cellular compartment or structure.

10.1128/mbio.00213-22.2 FIG S2 Multiple-gene alignment of Msmeg0070 orthologs from all 5 M. smegmatis isolates, using Clustal. The alignment uses the reannotated, upstream start site for mc^2^155 *Msmeg0070*. Download FIG S2, PDF file, 0.05 MB. Copyright © 2022 Clark et al. 2022 Clark et al. https://creativecommons.org/licenses/by/4.0/ This content is distributed under the terms of the Creative Commons Attribution 4.0 International license.

The reannotated start codon for *Msmeg0070* is 32 nt downstream of *Msmeg0069*, suggesting a possible operonic organization of *mid*. The amino acid similarity between Msmeg0069, Msmeg0070, and the N-terminal portion of Msmeg0071 is extremely low between all strains (with the notable exception of Jucho and mc^2^155). This portion of *esx1* is also the least conserved among different mycobacterial species, complicating the identification of homologs. Although putative Msmeg0070 homologs are widespread in environmental species, they have not been detected by sequence similarity searches in the M. tuberculosis complex. In some species, BLAST searches reveal that the tandem *Msmeg0069* and *Msmeg0070* orthologs are absent from the *esx1* locus but are colocated elsewhere in the genome, further suggesting that the encoded proteins are functionally associated. The sequence diversity observed among Msmeg0070 orthologs may be driven by the need for expressing the self-identity that determines DCT compatibility. Similarly, the diversity observed in Msmeg0069 and the N-terminal two-thirds of Msmeg0071 could reflect that they play similar roles in extracellular cell identity or that they interact (and coevolve) with Msmeg0070. While speculative, it is conceivable that polymorphic *mid* regions (Fig. 5) are evolutionary hot spots continually revised to distinguish close from distant kin in mycobacterial communities.

### Kin recognition, mycobacterial communication, and DCT.

Bacteria are known to use kin recognition to determine social groups in mixed populations, which generally results in some form of cooperative behavior between kin cells (22). This can be in the form of beneficial responses between kin to improve their competitiveness or antagonistic behavior against nonkin. While it is unclear whether DCT should be considered a congenial or antagonistic interaction, it is an activity between nonkin. Recognition is generally mediated by interactions between surface receptors and/or other extracellular molecules. The diversity of the recognition system increases the specificity of the process, allowing discrimination among closely related bacteria (conspecific strains), as observed in the polymorphic *mid* regions of M. smegmatis isolates. In the assays described here, DCT provides a direct readout of kin recognition, presumably mediated by Msmeg0070 at the cell surface. However, based on the broad precedent of functions established for many other bacteria (23–26), it is unlikely that mycobacterial kin recognition will be limited to DCT, especially for mycobacteria found in the environment.

Kin recognition is a form of cell-to-cell communication, and we assign this function to *Msmeg0070* within the *esx1* locus. Similarly, we had previously shown that direct cell-cell communication induces ESX-4 expression in the recipient to promote DCT and that the expression of ESX-4 is modulated by ESX-1 secretion from the donor (20). Thus, multiple processes, DCT, kin recognition, and ESX function, involve cell-cell communication. The functional intertwining of all three processes suggests that cell-cell signaling plays an important role in mycobacterial biology.

### Mid function is phenotypically, but not mechanistically, similar to surface exclusion.

The inhibition of DCT between two cells with identical *mid* regions is superficially similar to surface exclusion immunity encoded by conjugative plasmids (3). Surface and/or entry exclusion proteins are membrane-associated polymorphic proteins that prevent conjugal DNA transfer between cells containing the same plasmid. In surface exclusion, proteins create a physical barrier to cell adhesion and prevent stable mating-pair formation (27, 28). In contrast, entry exclusion proteins block the assembly of a functional transfer apparatus in the donor after mating-pair formation (29–31). However, despite the functional similarity of preventing conjugal DNA transfer, plasmid-encoded exclusion proteins are not homologous to Msmeg0070 (data not shown). Moreover, DCT and plasmid-mediated transfer are fundamentally different, and the distinct membrane structures encompassing mycobacteria and Gram-positive and Gram-negative bacteria make it extremely unlikely that entry exclusion and Mid proteins use related mechanisms. Thus, the mechanism of action of Msmeg0070 in identifying self is likely to be specific to Mycobacterium.

## MATERIALS AND METHODS

Bacteria were maintained in Trypticase soy broth with 0.05% Tween 80 containing antibiotics appropriate for each strain derivative (streptomycin at 200 μg/mL, kanamycin [Km] at 25 μg/mL, hygromycin at 25 μg/mL, apramycin at 25 μg/mL, or zeocin at 25 μg/mL) or on Trypticase soy agar plates. All cultures were grown at 37°C, except for mating assays, which were performed at 30°C. M. smegmatis strains were originally described previously (9, 13). Kanamycin-resistant derivatives of mc^2^155, Jucho, Rabinowitchi, and Nishi were created by integrating the kanamycin gene at the *attL5* locus to create MKD6, MKD22, MKD21, and MKD24, respectively. These strains were used in crosses with streptomycin-resistant derivatives of Jucho (J4) and mc^2^874 (MKD8) (9). Deletion mutants of *eccC*_b1_ and *esxBA* in mc^2^155 were created by recombineering, which replaced each gene with a cassette encoding zeomycin resistance (32). Mating assays were performed as previously described (9, 33), and data represent the averages from at least three independent assays.

### Genome sequencing.

Genomic DNA for sequencing was prepared as previously described (34, 35). Whole-genome DNA sequence analysis, using the PacBio platform, and *de novo* assembly were performed by the Institute for Genome Sciences (IGS), University of Maryland. Genomes were annotated using Prokka (36). The genomes of each strain were determined to be essentially collinear by SibeliaZ (14). SNVs were identified by Parsnp and visualized in Gingr for multiple-genome alignments (37).

Transconjugant genomes were sequenced using paired-end Illumina technology and assembled using shovill version 1.0.4 (https://github.com/tseemann/shovill) with a minimum contig length of 1 kb. Contigs were reoriented and reordered with RaGOO v1.11 (38). Sequences are available upon request. Genomic comparisons assigned SNVs in the transconjugant to either the donor or recipient to determine the location and extent of DNA inherited from each parent. Boundaries of recipient- and donor-derived segments were defined from nonoverlapping 500-bp sliding windows over the progeny genome to identify the origin of each fragment using identity scores derived from the FASTani algorithm (39). If a window had 100% identity to both parental genomes, this segment was assigned the same origin as that of the previous window (donor or recipient origin). Consecutive windows assigned to the same strain were concatenated to define the length of the inherited region and visualized using R (http://www.R-project.org).

### Genetic manipulation of the *mid* region and *Msmeg0070* in mc^2^155.

The *mid* region in mc^2^155 was deleted by the targeted placement of *loxP* sites between *Msmeg0068* and *Msmeg0069* and between *Msmeg0078* and *Msmeg0079*. Subsequently, the intervening region was deleted upon the expression of Cre that left a single *loxP* scar. The *Msmeg0069–0071 mid* genes in mc^2^155 were swapped with recipient orthologs by a two-step allele exchange system (40). The comparable recipient *mid*_MKD8_ region was amplified and cloned into the suicide vector derivative of pDB88 (40) that contained the *mid*_155_ flanking regions of the donor. These flanking regions provided the homology required for single-crossover, targeted recombination resulting in hygromycin-resistant (Hyg^r^) recombinants. This intermediate spontaneously resolved, and subsequent counterselection for the resolved product was facilitated by the loss of pDB88-encoded *galK-* and *sacB*-mediated conditional toxicity. The recombinant region was amplified by PCR, and its architecture was confirmed by DNA sequence analysis of the region.

The *Msmeg0070* and *Msmeg0071* genes were amplified by PCR and cloned into pGD6 (a Hyg^r^-encoding mycobacterial expression plasmid based on pSE100 [41]). All clones were sequence verified.

### Data availability.

Sequenced genomes of the natural isolates of M. smegmatis are available at the NCBI (see Table 1 for GenBank accession numbers and genome details). Sequences of oligonucleotide primers for mutagenesis and sequencing are available upon request.
