# Supplementary figures and images for: A Polymorphic Gene within the Mycobacterium smegmatis esx1 Locus Determines Mycobacterial Self-Identity and Conjugal Compatibility
Source: mBio. 2022 Mar 17;13(2):e00213-22. doi: 10.1128/mbio.00213-22 (PMC9040860; doi:10.1128/mbio.00213-22)

JxM1

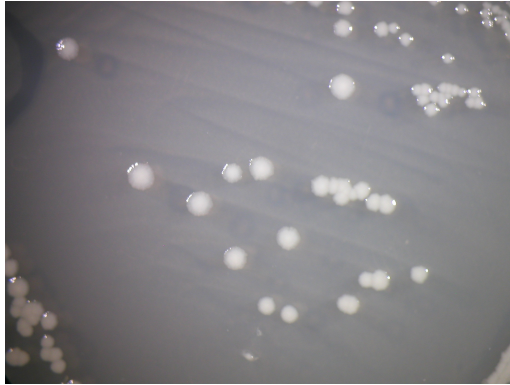

JxM4

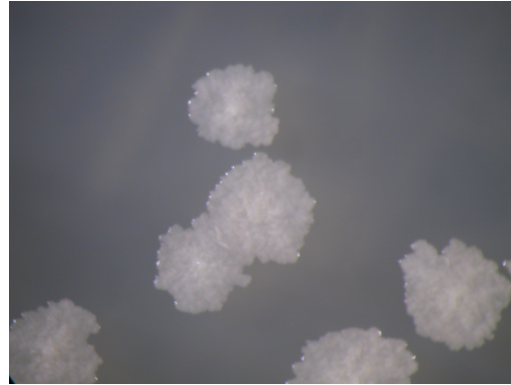

JxM2

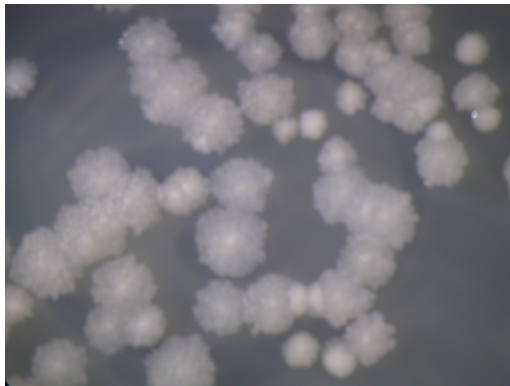

JxM6

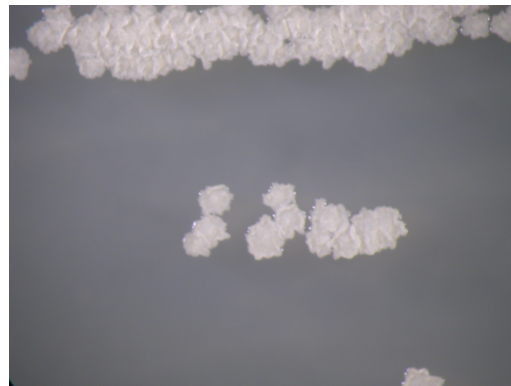

JxM3

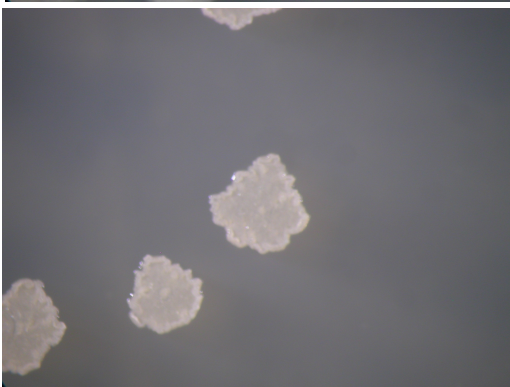

JxM8

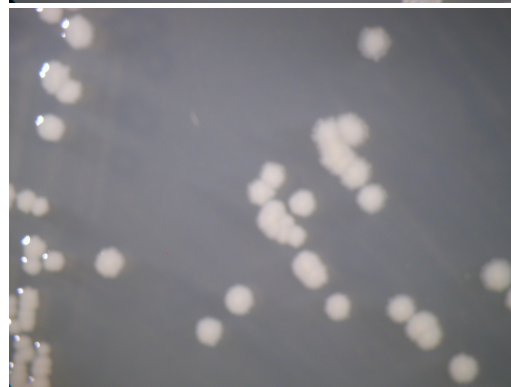

Supplement: FIG S1 [file mbio.00213-22-sf001.pdf]
