# Supplementary material for: A Polymorphic Gene within the Mycobacterium smegmatis esx1 Locus Determines Mycobacterial Self-Identity and Conjugal Compatibility
Source: mBio. 2022 Mar 17;13(2):e00213-22. doi: 10.1128/mbio.00213-22 (PMC9040860; doi:10.1128/mbio.00213-22)
Supplement: TABLE S2 [file mbio.00213-22-st002.docx]

| Strain | mc^2^155 | Jucho | MKD8 | Nishi | Rabinowitchi |
| --- | --- | --- | --- | --- | --- |
| mc^2^155 | 0 | 2 | 464 | 335 | 365 |
| Jucho |  | 0 | 464 | 335 | 365 |
| MKD8 |  |  | 0 | 230 | 248 |
| Nishi |  |  |  | 0 | 147 |
| Rabinowitchi |  |  |  |  | 0 |
