# Supplementary material for: A Polymorphic Gene within the Mycobacterium smegmatis esx1 Locus Determines Mycobacterial Self-Identity and Conjugal Compatibility
Source: mBio. 2022 Mar 17;13(2):e00213-22. doi: 10.1128/mbio.00213-22 (PMC9040860; doi:10.1128/mbio.00213-22)
Supplement: FIG S2 [file mbio.00213-22-sf002.pdf]

```
             1
MKD8         MTTPPGPE-- -----IPPPH QGGF------ ---------- ------YSAG HHPQRPWPET  PP-PKTRGG
Rabinowitchi MTLPPPPNTP PPNGASPPPP FGGS------ ---------- ------GPYP QSLHQPWPQQ  PS-PRRSGT
mc2155       MTLPPPPGSF G-----QQPP LGGHSGGGSP ESWPQYPGGP TSPPPAGPPP WGPQPQWANG  PTPPNGGGK
Jucho        MTLPPPPGSF G-----QQPP LGGHSGGGSP ESWPQYPGGP TSPPPAGPPP WGPQPQWANG  PTPPNGGGK
Nishi        ---------- ---------- ---------- ---------- ---------- ----------  ---------

            71
MKD8         VKWMLGAVAL LAVVGVTVAV TLA--VTGKD KRDAIPPGSG VSGSPTASDI A SADDSGPV SVITEDPTCA
Rabinowitchi WKWVLAIVAL LAVVGVTAAV TVT--VTGEP TGGGLPSAST PQAVPSDSEI A SAEDSGGV SVITDDPTCA
mc2155       VRWMLGGLAV V--LAIALAV VVTVLVVRPD AENG---SKD EKTGGPASGF A SENDDGPV SIITDDPTCD
Jucho        VRWMLGGLAV V--LAIALAV VVTVLVVRPD AENG---SKD EKTGGPASGF A SENDDGPV SIITDDPTCD
Nishi        MNLPPPRVSV TLAIALAVAV AVAVLVVRSD GEDR------ ----PSESAY A SAGDTDPV NLITEEPTCA

           141
MKD8         AQGPILETFA AQQ-SQLWVE RDPALGRESW SPELRADYEK VG KAMRTAA DQVAQLAKIT PHRAMRELYE
Rabinowitchi PQRPIVSTLA AKT-NAGWDK RDPEVPASDW TPEIRAQYNE VA AAMRDAA DQFVQLAKLT PHRVMREIYE
mc2155       GWARITREYN AESTAVRWAE RDASIPANAW TPEQRDMYNT MA KAMTTAA DHTEALIKQT PHRAMRELYQ
Jucho        GWARITREYN AESTAVRWAE RDASIPANAW TPEQRDMYNT MA KAMTTAA DHTEALIKQT PHRAMRELYQ
Nishi        PWMSVVEEFT REAAAVNWQG RRADVPASAW TPAERATYDT VG KAMARMA DRAPKFAQQT PHRVMREPYE

           211
MKD8         QFIAYARAYA DNIPNYTPPT DNLARVAVTA ADA ISYICA AVSYGSAAAR APLVENRPAP TNVAPLGNPS
Rabinowitchi QFIAYARAYA ASVPNYTPGD DYSARFAVGA AEA ISRICA AIDYGSAGAR APLVKVSEPP AEIAPVGDAN
mc2155       QFSAYVHVFV PLIPAYVPEN NRFVPVINAL ANS ATDICT AIEFRSASTF AGRIPSVDPP SRLASTSGAD
Jucho        QFSAYVHVFV PLIPAYVPEN NRFVPVINAL ANS ATDICT AIEFRSASTF AGRIPSVDPP SRLASTSGAD
Nishi        QMVAYTRAFV DRIPTYVPED NKLLSASSAA GSS ISNICG AIAYGTARDV TPLLAAAPVP ADPVTPQEYS

           281
MKD8         EPERFLTAPN PVCGEWSSVL NAFQ TDTTE WLKTDPDISS SQWSIEQKQI NENVIPIMKR FANQLYLLGK
Rabinowitchi APVRFLSEPN AVCRDWNLAL MQFQ DETAE WRNTDPKIPA GEWTPEQKRL NVEVAPVMRR FATQLRSLGN
mc2155       ASDLFLGDGN PVCADLASAV VAFD EQTRA WQALDPKLPA AEWSPDHRAV MDDVAPVMSA NADNLERLGR
Jucho        ASDLFLGDGN PVCADLASAV VAFD EQTRA WQALDPKLPA AEWSPDHRAV MDDVAPVMSA NADNLERLGR
Nishi        EPETLVTQSN PACPDWSAEA DEFS AKSAD WLATSKRVPA AEWTPQQKAV HETVAPMMAA NADKLEQLGR

           351
MKD8         DSGNPTFRDI ADLSV QYRL AYVAAIPTYT PADKYLANAS IRLATMANVA CRAAAD
Rabinowitchi SSGNPVLADF ANLSA QYRS ALEKALPTYV PADDYLAGVS ARLNGMVNSA CQAVSA
mc2155       ASDNAIVEDF TVLAA QYQR GYVEAIPTYS SADNVLWQVV ASLVKAVNSG CKAS--
Jucho        ASDNAIVEDF TVLAA QYQR GYVEAIPTYS SADNVLWQVV ASLVKAVNSG CKAS--
Nishi        QSGDPVLEDV AALQR STDG PSSSHCPTTR A-PTGCWRPR RRNW----SG ------
```
